# Supplementary material for: “Obesity, metabolic abnormalities and low-grade inflammation: differencial associations with subclinical atherosclerosis”
Source: Front Cardiovasc Med. 2025 Sep 15;12:1607399. doi: 10.3389/fcvm.2025.1607399 (PMC12477694; doi:10.3389/fcvm.2025.1607399)

SUPPLEMENTARY MATERIAL

Supplementary Table 1: Comparative analysis between NOBE and OBE subjects

VARIABLE	NOBE (n=4640)	OBE (n=2095)	P
**Age**, years (median±IQR)	52 (45-60)	51 (44-59)	0.0680*
**Male sex**, %	49.3	73.8	< 0.0001#
**BMI**, kg/m2 (median±IQR)	25.2 (22.6-27.4)	33.7 (31.7-36.9)	< 0.0001*
**SBP**, mm Hg (median±IQR)	123 (113-134)	130 (122-141)	< 0.0001*
**DBP**, mm Hg (median±IQR)	80 (73-87)	85 (78-92)	< 0.0001*
**GLU**, mg/dL (median±IQR)	95.0 (89-102)	100 (93-108)	< 0.0001*
**HDL-C**, mg/dL (median±IQR)	54.0 (46-64)	45.0 (39-53)	< 0.0001*
**TG**, mg/dL (median±IQR)	98.0 (73-135)	131 (97-179)	< 0.0001*
**NEU**, x10^9/L (median/IQR)	3.50 (2.83-4.25)	3.90 (3.18-4.72)	< 0.0001*
**DBT**, %	4.20	10.5	< 0.0001#
**Smoking Habit**, %	14.0	12.3	0.0590#

Note: *Mann Whitney test.
#Chi-Square test

Note: Age, anthropometrics, metabolic, inflammatory variables and smoking habit in Non-obese vs. Obese subjects

Supplementary Table 2: Comparative analysis between MH and MU groups

VARIABLE	MH (n=1782)	MU (n=4953)	P
**Age,** years (median±IQR)	48.0 (42-55)	53.0 (45-61)	< 0.0001*
**Male sex**, %	39.7	63.5	< 0.0001#
**BMI**, kg/m^2 (median±IQR)	24.2 (21.3-27.1)	28.4 (25.2-32.4)	< 0.0001*
**SBP**, mm Hg (median± IQR)	114 (108-121)	131 (122-140)	< 0.0001*
**DBP**, mm Hg (median±IQR)	73 (68-78)	85 (78-91)	< 0.0001*
**GLU**, mg/dL (median±IQR)	92.0 (87-97)	98.0 (92-106)	< 0.0001*
**HDL-C**, mg/dL (median±IQR)	59.0 (52-69)	48.0 (41-57)	< 0.0001*
**TG**, mg/dL (median±IQR)	81.0 (64-104)	122 (88-171)	< 0.0001*
**NEU**, x10^9/L (median/IQR)	3.30 (2.69-4.06)	3.74 (3,06-4.54)	< 0.0001*
**DBT**, %	0.00	9.54	< 0.0002#
**Smoking Habit**, %	13.4	13.8	0.1920#

Note: *Mann Whitney test.
#Chi-Square test

Note: Age, anthropometrics, metabolic, inflammatory variables and smoking habit in Metabolic Healthy vs. Metabolic Unhealthy groups.

Supplementary Table 3: Independent variables associated to ATS presence in logistic regression model 2

Variable	Unadjusted OR (95% CI)	P values	Adjusted OR (95% CI)	P values
**Age**	1.12 (1.12-1.13)	<0.0001	1.13 (1.12-1.14)	<0.0001
**Male sex**	2.50 (2.30-2.70)	<0.0001	3.33 (2.92-3.79)	<0.0001
**Sedentary habit**	1.41 (1.27-1.56)	<0.0001	1.13 (0,99-1.29)	0.0730
**Smoking**	1.57 (1.40-1.80)	<0.0001	2.16 (1.81-2.59)	<0.0001
**CVD-FH**	0.98 (0.86-1.12)	0.7560	1.10 (0.93-1.29)	0.2764
**NEU**	1.09 (1.04-1.13)	<0.0001	1.08 (1.03-1.14)	0.0018
**OBE**	1.23 (1.11-1.37)	0.0001	0.88 (0.77-1.01)	0.0831
**MA**	2.98 (2.66-3.33)	<0.0001	1.82 (1.58-2.10)	<0.0001

Model 2:$P < 0.0001$, R^2 =0,42 (Nagelkerke), Hosmer & Lemeshow test P= 0.14; AUC (ROC)= 0.83(95% CI 0.82-0.84)

Note: In this logistic regression model atherosclerosis was defined as the dependent variable, while Obesity, Metabolic Abnormalities and Neutrophil count were explored as independent predictors of atherosclerosis adjusted by Age, Male Sex, Sedentarism, Smoking Habit and Family History of Cardiovascular Disease.

Supplementary Figure 1: Maximal carotid inthima-media thickness differences among MHNO, MHO, MUNO and MUO groups.

Note: The Kruskal–Wallis test indicated a statistically significant difference in median CIMT values across groups (H = 36.273, df = 3, *p* < 0.001). The highest CIMT was observed in the MUO group, followed by MUNO and MHO groups. The lowest CIMT values were found in the MHNO group. CIMT values were expressed as median/IQR (CI 25-75%).

Supplementary Figure 2: Prevalence of subclinical ATS between MH and MU groups

Note: The prevalence of ATS in Metabolic Healthy subjects was 33.3% vs. 59.7% in Metabolic Unhealthy subjects; *p* <0.0001 (Chi Square Test).

Supplementary Figure 3: ROC curve of logistic regression model 1

Supplementary Figure 4: ROC curve of logistic regression model 2

Supplementary Figure 1: Maximal Carotid Inthima-Media Thickness differences among MHNO, MHO, MUNO and MUO groups.

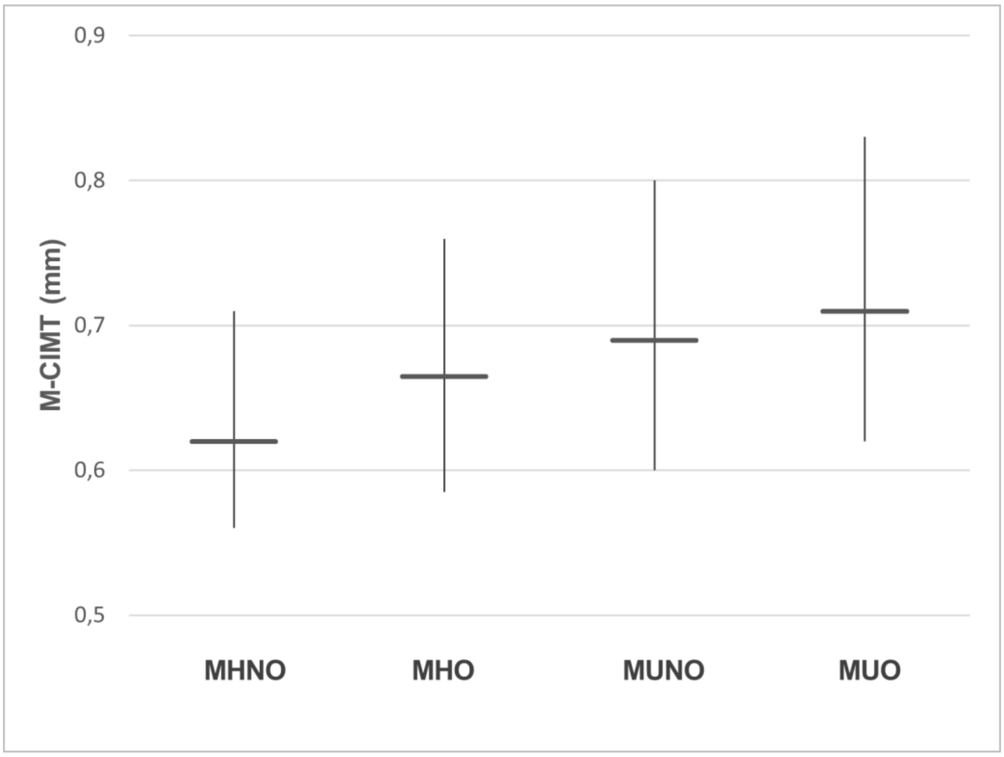

Supplementary Figure 2: Prevalence of subclinical ATS between MH and MU groups

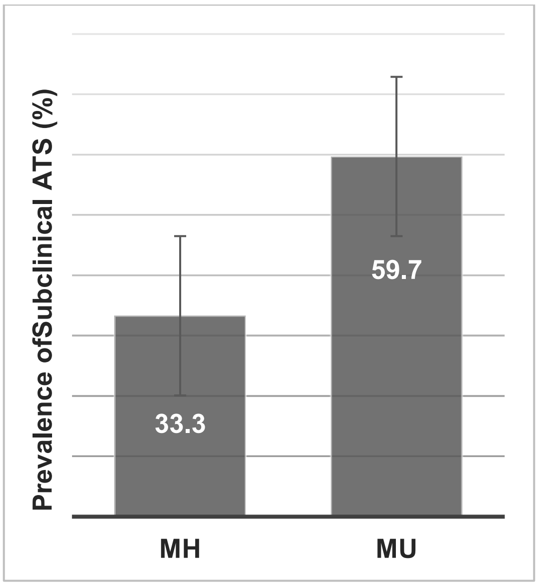

Chi Square Test. *p* <0.0001(MH vs. MU groups)

Supplementary Figure 3: ROC curve of logistic regression model 1

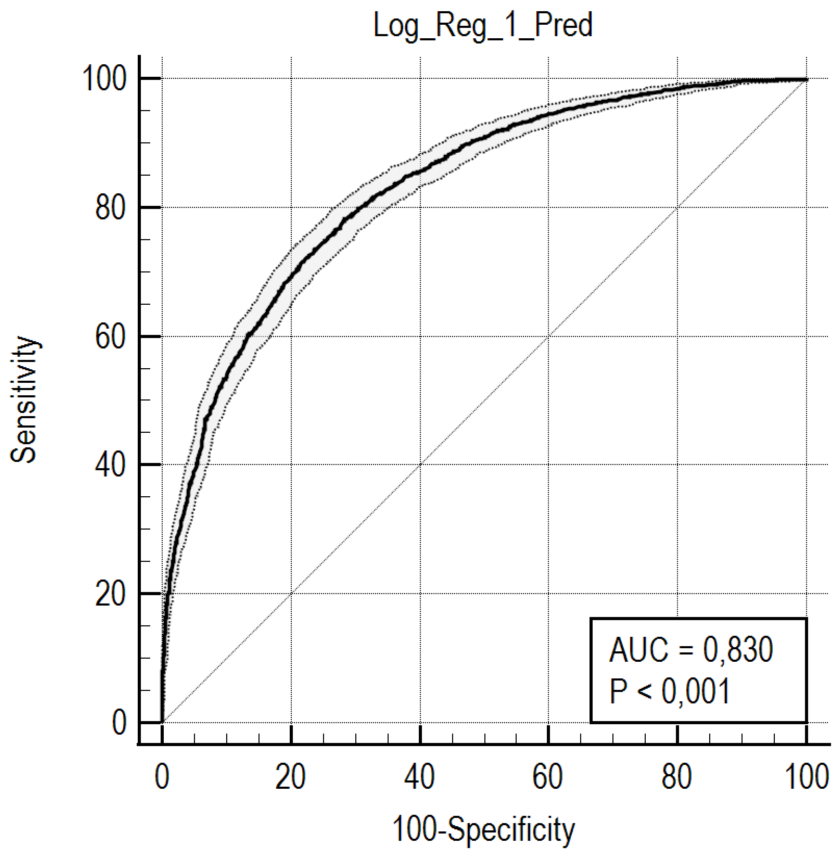

Supplementary Figure 4: ROC curve of logistic regression model 2

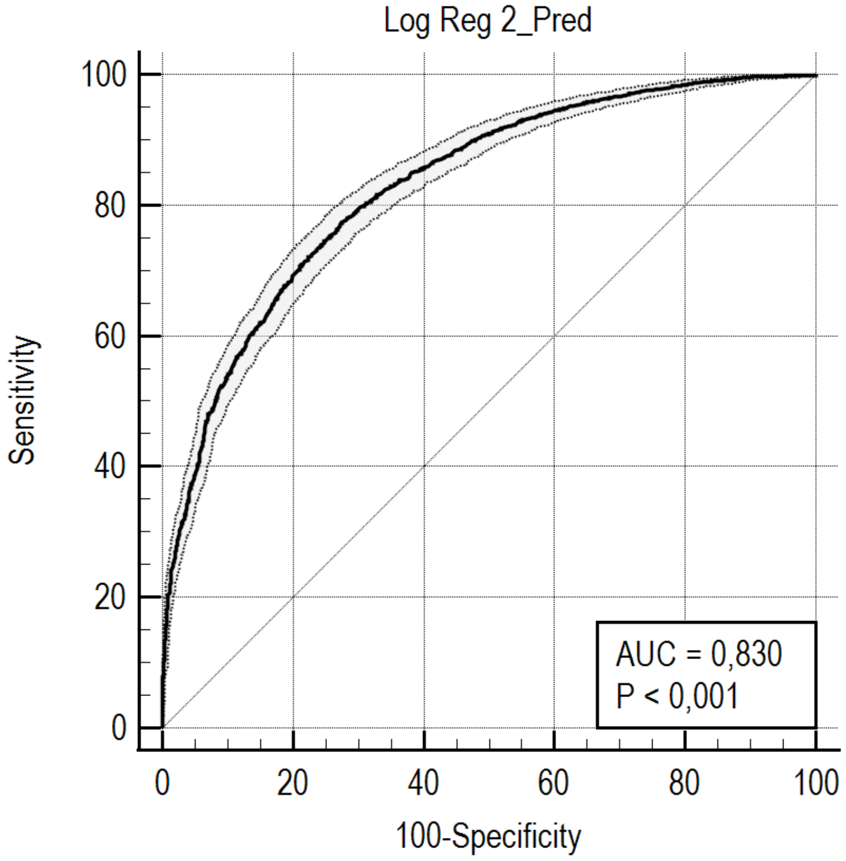

Supplement: Supplementary file 1 [file Datasheet1.pdf]
